# A conformation-specific antibody against oligomeric β-amyloid restores neuronal integrity in a mouse model of Alzheimer's disease

**DOI:** 10.1074/jbc.RA120.015327

**Published:** 2021-01-09

**Authors:** Ping He, Philip Schulz, Michael R. Sierks

**Affiliations:** Department of Chemical Engineering, Arizona State University, Tempe, Arizona, USA

**Keywords:** single-chain antibody, oligomeric beta amyloid, neuron, Alzheimer's disease, transgenic mice, Aβ, amyloid β, AD, Alzheimer's disease, ApoB, apolipoprotein B, APP, amyloid precursor protein, BBB, blood brain barrier, DCX, doublecortin, GFAP, glial fibrillary acidic protein, MAP2, microtubule-associated protein 2, ND, nondementia, rAAV, recombinant human adeno-associated virus, scFv, single chain antibody variable domain fragment, SYP, synaptophysin

## Abstract

Conformationally distinct aggregates of the amyloid β (Aβ) peptide accumulate in brains of patients with Alzheimer's disease (AD), but the roles of the different aggregates in disease progression are not clear. We previously isolated two single-chain variable domain antibody fragments (scFvs), C6T and A4, that selectively bind different toxic conformational variants of oligomeric Aβ. Here, we utilize these scFvs to localize the presence of these Aβ variants in human AD brain and to demonstrate their potential as therapeutic agents for treating AD. Both A4 and C6T label oligomeric Aβ in extracellular amyloid plaques, whereas C6T also labels intracellular oligomeric Aβ in human AD brain tissue and in an AD mouse model. For therapeutic studies, the A4 and C6T scFvs were expressed in the AD mice by viral infection of liver cells. The scFvs were administered at 2 months of age, and mice sacrificed at 9 months. The scFvs contained a peptide tag to facilitate transport across the blood brain barrier. While treatment with C6T only slightly decreased Aβ deposits and plaque-associated inflammation, it restored neuronal integrity to WT levels, significantly promoted growth of new neurons, and impressively rescued survival rates to WT levels. Treatment with A4 on the other hand significantly decreased Aβ deposits but did not significantly decrease neuroinflammation or promote neuronal integrity, neurogenesis, or survival rate. These results suggest that the specific Aβ conformation targeted in therapeutic applications greatly affects the outcome, and the location of the targeted Aβ variants may also play a critical factor.

Alzheimer's disease (AD) is an age-related neurodegenerative disorder characterized by the presence of amyloid β (Aβ) plaques (1, 2). Human AD plaques contain a wide variety of different soluble oligomeric Aβ species (3), some of which have been postulated as the toxic species responsible for the pathogenesis and spread of AD (4, 5, 6, 7, 8). The hypothesis that oligomeric Aβ species are responsible for AD pathogenesis rather than the fibrillar amyloid plaques is supported by many studies including a mouse model engineered to express oligomeric Aβ but not plaques (amyloid precursor protein [APP]^E693Q^) (6, 9), where mice engineered to convert oligomers into plaques (APP^E693Q^/PS1ΔE9) were not impaired to a greater extent than the mice generating only oligomeric Aβ (10).

A variety of different oligomeric Aβ conformations have been detected using atomic force microscopy imaging (11, 12, 13, 14, 15, 16, 17, 18). Atomic force microscopy can be used to estimate the number of monomeric Aβ units in each oligomeric assembly based on the particle size. For example, particles with an average diameter of ∼10 nm corresponded to high–molecular-weight oligomers, and ones with average diameter 3 to 6 nm corresponded to low–molecular-weight oligomers (12, 13, 15, 16, 17, 18, 19). The different aggregate structures could have different consequences as the high–molecular-weight Aβ oligomers accelerated Aβ fibrillogenesis, whereas other complexes have been shown to create pores that could facilitate disintegration of membrane structures (19).

Since distinct oligomeric Aβ variants can induce toxicity by different mechanisms, it has been suggested that different treatments to inhibit the toxicity of the different Aβ species may be needed (19). Previously, we generated two single-chain antibody variable domain fragments (scFvs), C6T and A4, that bind two conformationally distinct toxic oligomeric Aβ species (16, 17, 18). The A4 scFv (17) binds an oligomeric Aβ variant that can be generated synthetically by incubating monomeric Aβ in a test tube. In contrast, the C6T scFv (18) binds an oligomeric Aβ variant isolated from human brain tissue but does not bind synthetically generated Aβ variants. The oligomeric Aβ species recognized by C6T is considered to be a cell-derived oligomeric Aβ species (20). Since both these scFvs reduced toxicity of Aβ in *in vitro* assays (17, 18, 21), the scFvs are promising therapeutics to selectively target different Aβ variants. The C6T and A4 scFvs were administered to an APP/PS1 mouse model of AD (Mutant Mouse Resource and Research Center 34832) using viral vectors to express the scFvs essentially as described previously (22). We used a serotype AAV2/8 vector to express the gene construct in liver cells of infected mice. The low-density lipoprotein receptor–binding domain of apolipoprotein B (ApoB) was added to the scFv constructs to facilitate transport across the blood brain barrier (BBB) (22, 23, 24). Viral vectors were administered to mice at 2 months of age, and mice were sacrificed and brains harvested at 9 months of age.

## Results

### Localization of oligomeric Aβ variants by C6T and A4 scFvs in human AD brains

**Figure 1:**
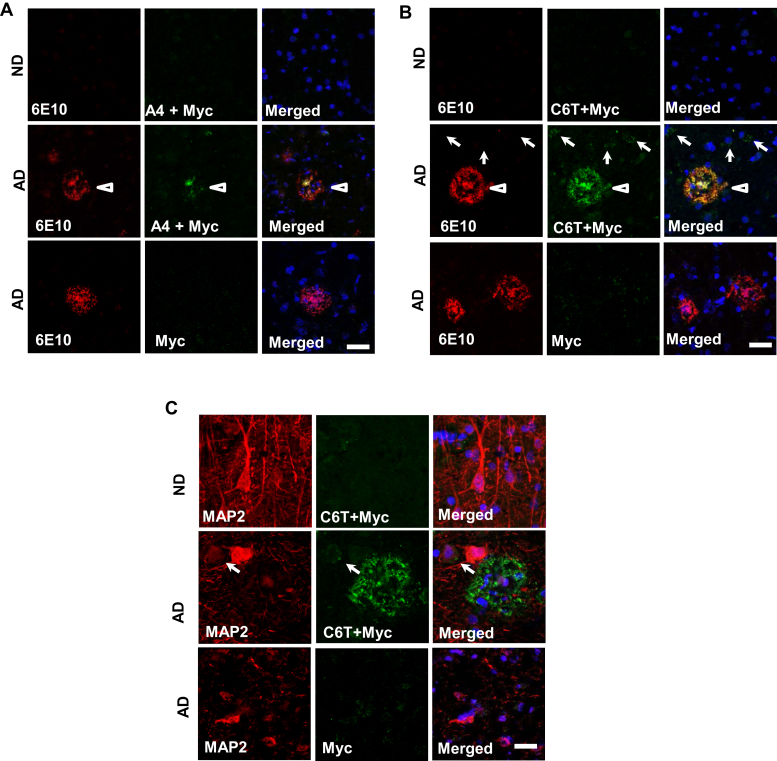
**A4 and C6T immunopositive structures in postmortem human brain tissue.** Aβ aggregates were stained using an antibody against Aβ_1–17_ (clone 6E10, *red*). Little staining of A4 and C6T as well as 6E10 was observed in the human brains with nondementia (ND). Counter staining by 4',6-diamidino-2-phenylindole. *A*, the oligomeric Aβ variants recognized by A4 scFv (*green*), together with Aβ aggregates stained by antibody 6E10 (*red*, *arrowhead*), in AD brain tissue. *B*, the oligomeric Aβ variants recognized by C6T scFv (*green*), together with Aβ plaques shown by 6E10 (*red*, *arrowhead*), as well as C6T-positive staining around the nuclei (*arrow*), in AD brain tissue. *C*, the extracellular and intracellular aggregates that C6T recognized were observed (*green*) in AD but not ND brain tissue. A control using secondary antibody against cMyc without the primary scFv is also shown. The neuronal cells were stained by specific antibody against MAP2 (*red*, *arrow*). The bars represent 20 μm. MAP2, microtubule-associated protein 2.

We previously isolated two scFv fragments, A4 and C6T, against two conformationally distinct oligomeric Aβ species, one synthetically generated, and one human AD brain derived (Table 1) (17, 18, 21). We showed that the A4- and C6T-recognized oligomeric Aβ variants could both be detected in human brain tissue (18), cerebral spinal fluid (25), as well as sera samples (25, 26). Here, we immunostained brain sections of postmortem human AD and control cases with purified A4 and C6T scFvs. Results showed little expression of A4- and C6T-recognized oligomeric variants in the age-matched nondementia (ND) brains (Fig. 1, *A* and *B*). In AD brain tissue, the A4-positive variants were observed to be localized together with Aβ plaques, which were identified using a monoclonal antibody against Aβ_1–17_ (6E10; Fig. 1*A*). Similar to A4 staining, the C6T-recognized oligomers also colocalized with Aβ aggregates (Fig. 1*B*). In addition, we found C6T-positive structures around the nuclei (Fig. 1*B*). In order to further confirm whether C6T stained Aβ variants within neurons, we double-stained C6T with neuronal marker microtubule-associated protein 2 (MAP2) and observed perinuclear cytoplasmic C6T-immunoreactive inclusions within neurons labeled by MAP2 (Fig. 1*C*). We further demonstrated that C6T stains intracellularly generated Aβ variants in a mammalian cell line overexpressing Aβ (Fig. S1).

**Table 1:** Comparison of targets identified by Aβ conformation-specific scFvs A4 and C6T

Characteristics	scFv-A4 (Zameer *et al.*, 2008 (17); Kasturirangan *et al.*, 2012 (16))	scFv-C6T (Kasturirangan *et al.*, 2013 (18))
Antigen epitope	Synthetic Aβ1-42 aggregate	Human-derived oligomeric Aβ
Phage library	Tomlinson I+J (Winter *et al.*, 1994)	Sheets (Sheets *et al.*, 1998)
Aβ aggregate height	∼3.6 nm	∼2.1 nm
Aβ aggregate diameter	∼10 nm	∼5 nm
Aβ fragment number	∼12-mers (dodecamer)	∼3–4 mers (tri/tetramer)

The aggregate sizes were measured with the height of size under atomic force microscopy and expressed as an averaged size by scale nanometer.

### Oligomeric variants recognized C6T and A4 in the brains of APP/PS1 mice

**Figure 2:**
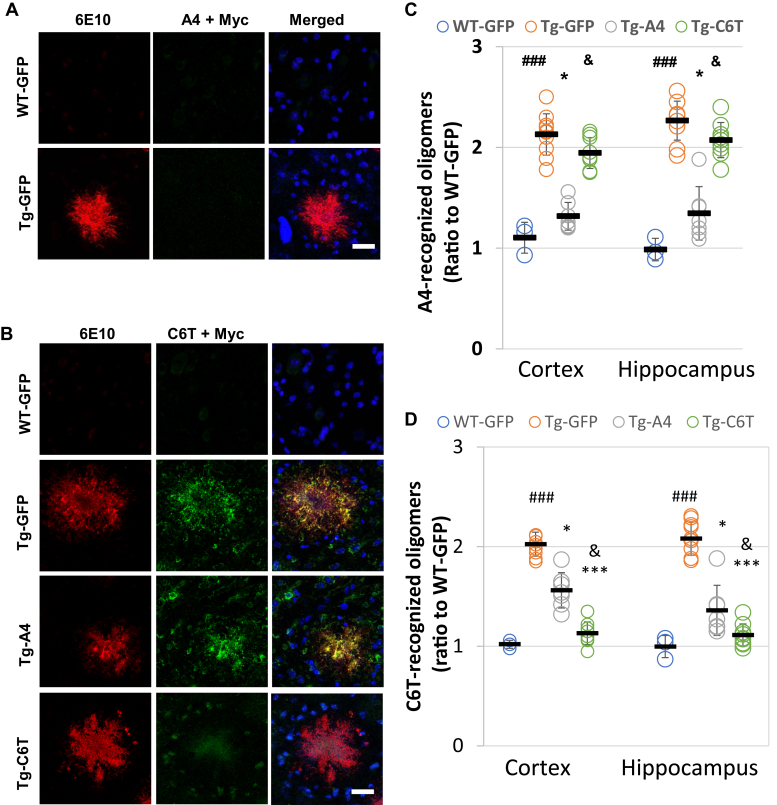
**Localization and levels of A4- and C6T-recognized Aβ structures in brain tissue from APP/PS1 mice.** Aβ accumulation was stained by a specific antibody against Aβ_1–17_ (6E10, *red*). Counter staining by 4',6-diamidino-2-phenylindole. *A*, staining with purified A4 scFv in the APP/PS1 mice brain tissue. *B*, the staining of C6T-recognized oligomeric Aβ (*green*) and Aβ aggregates (6E10, *red*) in WT-GFP, Tg-GFP, Tg-A4, and Tg-C6T mouse brain tissue and negative staining of A4 and C6T, as well as 6E10 in the brains of WT mice. The bars represent 50 μm. *C*, levels of oligomeric Aβ reactive with A4 scFv following treatment with rAAV-GFP, rAAV-A4, and rAAV-C6T. *D*, levels of oligomeric Aβ reactive with C6T scFv following treatment with rAAV-GFP, rAAV-A4, and rAAV-C6T. All data were expressed as fold increase relative to WT-GFP mice in the cortex and hippocampus. The quantitative assay was performed with the mice of WT-GFP (n = 3), Tg-GFP (n = 10), Tg-A4 (n = 7), and Tg-C6T (n = 9). ^###^*p* < 0.001 to age-matched WT-GFP mice. ∗*p* < 0.05 and ∗∗∗*p* < 0.001 to littermate Tg-GFP vehicle mice, and ^&^*p* < 0.05 to littermate Tg-A4 mice.

Following staining on human brain sections, similar studies were performed on brain slices of APP/PS1 mice, a mouse model of AD. We did not find any expression of the oligomeric Aβ variants recognized by C6T and A4 in the brains of WT mice (Fig. 2, *A* and *B*), similar to our observations with ND human brain tissue (Fig. 1, *A* and *B*). While A4 staining was observed in human AD brain tissue, we did not see expression of A4-recognized Aβ variants in the brains of APP/PS1 mice (Fig. 2*A*). However, similar to the results observed in the human AD brain tissue, we also observed colocalization of C6T-positive oligomers with Aβ plaques marked by specific Aβ antibody 6E10 as well as C6T-positive staining inclusions (Fig. 2*B*). Furthermore, we observed that the brain sections of mice treated with the recombinant human adeno-associated virus (rAAV)-C6T showed greatly reduced staining of C6T-immunoreactive structures (Fig. 2*B*). The levels of the A4 and C6T recognized that oligomeric Aβ variants in mouse brain homogenates were determined by ELISA. The vehicle-treated APP/PS1 mice showed significant increases in the levels of both A4- and C6T-specific oligomeric variants compared with WT (Fig. 2, *C* and *D*; ^###^*p* < 0.001), similar to previous results (22). In the mice treated with either rAAV-C6T or rAAV-A4, the levels of both A4-reactive oligomeric variants (Fig. 2*C*; ∗*p* < 0.05) and C6T-reactive variants (Fig. 2*D*; ∗*p* < 0.05) were lowered compared with the vehicle-treated mice.

### Both C6T and A4 decrease Aβ deposits in APP/PS1 mice

**Figure 3:**
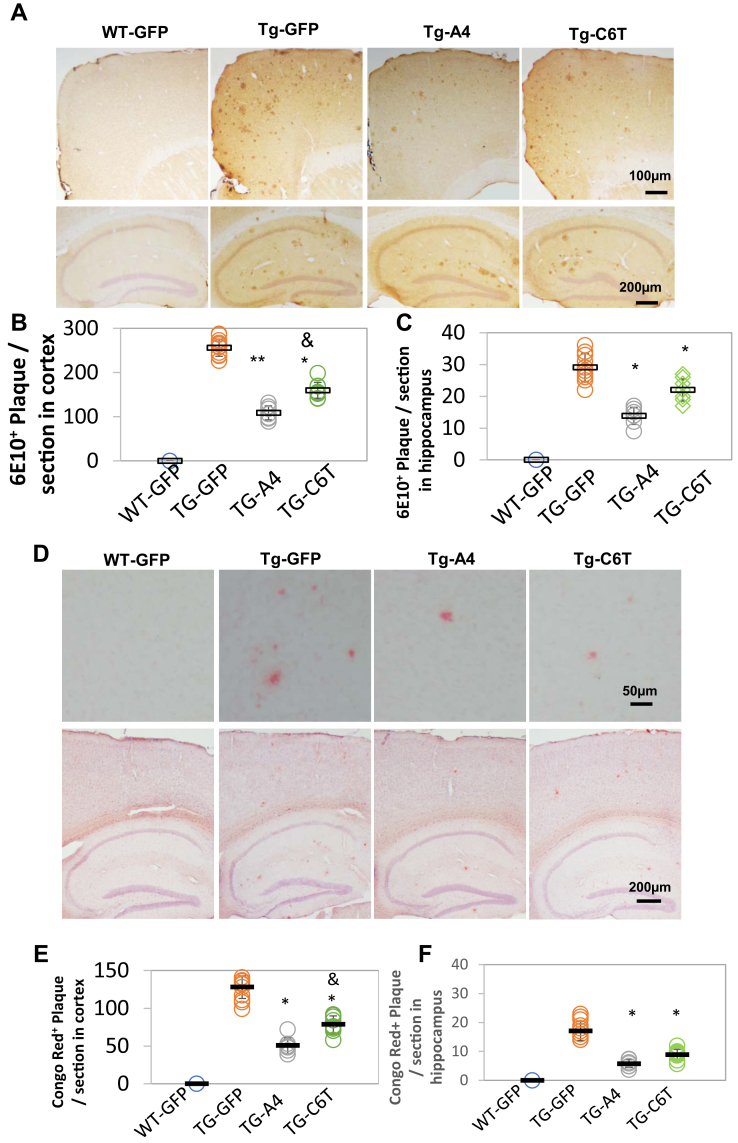
**Aβ staining in APP/PS1 mouse brain tissue.***A*, Aβ deposits were stained using anti-Aβ antibody 6E10 staining in the cortex (*top*) and the hippocampus (*bottom*) following the treatment with rAAV-GFP, rAAV-A4, and rAAV-C6T. *B* and *C*, the number of 6E10-positive deposits was counted and averaged per section in the cortex (*B*) and the hippocampus (*C*) of mice treated with rAAV-GFP, rAAV-A4, and rAAV-C6T. *D*, fibrillar deposits were verified by Congo red staining in the cortex (*top*) and the hippocampus (*bottom*). *E* and *F*, the number of fibrillar deposits was counted and averaged per section in the cortex (*E*) and the hippocampus (*F*). Nuclei were counterstained with hematoxylin. WT-GFP (n = 3), Tg-GFP (n = 15), Tg-A4 (n = 7), and Tg-C6T (n = 9). ∗*p* < 0.05 and ∗∗*p* < 0.01 to Tg-GFP mice and ^&^*p* < 0.05 to littermate Tg-A4 mice.

Aβ accumulation develops into plaques in the parenchyma (27). Immunostaining showed amyloid accumulation 6E10-specific antibody in the brains of APP/PS1 mice but not in WT mice (Fig. 3*A*). The number of plaques significantly decreased in the cortex (Fig. 3*B*; ∗∗*p* < 0.01, ∗*p* < 0.05) and the hippocampus (Fig. 3*C*; ∗*p* < 0.05) of mice receiving either rAAV-A4 and rAAV-C6T compared with the littermate GFP vehicle group. Interestingly, the number of 6E10-staining plaques in the cortex decreased significantly more in the rAAV-A4–treated mice compared with the rAAV-C6T–treated mice (Fig. 3*B*; ^&^*p* < 0.05) and were lower, though not statistically significantly, in the hippocampus as well.

The plaques are considered to be composed of a tangle of regularly ordered amyloid fibrillar aggregates (28). The histological staining using Congo red dye showed water-insoluble fibrillar plaques in the brains of APP/PS1 mice but not in WT mice (Fig. 3*D*). A significant decrease in the number of Congo red deposits was observed in both the cortex (Fig. 3*E*; ∗*p* < 0.05) and hippocampus (Fig. 3*F*; ∗*p* < 0.05) in brain tissue of mice treated with rAAV-A4 and rAAV-C6T compared with littermate GFP vehicle-treated mice. Similar to the results obtained with the 6E10-labeled plaques (Fig. 3B), a significant decrease in the number of Congo red structures was observed in the cortex in mice treated with rAAV-A4 compared with rAAV-C6T (Fig. 3*F*; ^&^*p* < 0.05), and levels were also lower in the hippocampus.

### Both A4 and C6T decrease microgliosis and gliosis in APP/PS1 mice

**Figure 4:**
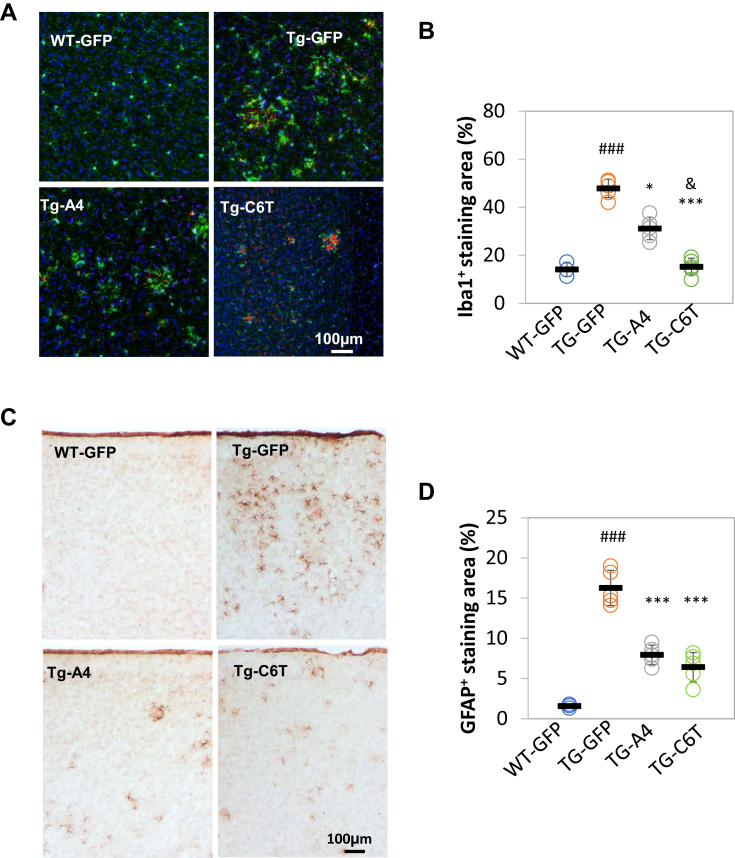
**Gliosis in response to Aβ in APP/PS1 mouse brain tissue.***A*, the immune cell microglia were immunostained by specific antibody against Iba1 (*green*). Aβ deposits were visualized by antibody against Aβ_1–17_ (clone: 6E10, *red*). Counter stain by 4',6-diamidino-2-phenylindole (*blue*). *B*, positive staining of microglia was expressed as a percentage of total area. *C*, reactive astrocytes were visualized using an antibody against GFAP. The fibrillar deposits were visualized by Congo red staining. Cell nuclei were counterstained by hematoxylin. *D*, positive staining of reactive astrocytes was expressed as a percentage of total area. WT-GFP (n = 3), Tg-GFP (n = 5), Tg-A4 (n = 5), and Tg-C6T (n = 5). ^###^*p* < 0.001 to age-matched WT-GFP mice, ∗*p* < 0.05 and ∗∗∗*p* < 0.001 to littermate Tg-GFP group, and ^&^*p* < 0.05 to littermate Tg-A4 group. GFAP, glial fibrillary acidic protein.

High levels of Aβ burden may be cytotoxic and activate glial cells leading to altered inflammatory responses (27, 29). Since microglia activation is a response to the presence of toxic substrates (27, 29), we can assess the effects of C6T and A4 treatments on inflammation through microglial activation. The double immunostaining was performed using antibody Iba1, a microglial marker, and 6E10 as an Aβ marker. Representative images showed a correlation of Iba1-positive cells with 6E10-labeling Aβ in the cortex of WT-GFP, Tg-GFP, Tg-A4, and Tg-C6T (Fig. 4*A*). Compared with age-matched WT-GFP vehicle group, the area of reactive microglia was greatly elevated in Tg-GFP vehicle mice (Fig. 4*B*; ^###^*p* < 0.001). In the rAAV-C6T and rAAV-A4 treated groups, there was a significant reduction in the area of reactive microglial compared with littermate GFP transgenic vehicle group (Fig. 4*B*; ∗∗∗*p* < 0.001, ∗*p* < 0.05). Interestingly, mice treated with rAAV-A4 showed reduced plaque formation compared with mice treated with rAAV-C6T, whereas mice treated with rAAV-C6T showed significantly reduced reactive microglia compared with the rAAV-A4 group (Fig. 4*B*; ^&^*p* < 0.05).

### Both A4 and C6T decrease astrocyte activation in APP/PS1 mice

Glial fibrillary acidic protein (GFAP) is another marker of neurodegeneration and neuronal injury (30). Immunostaining was performed using an antibody against GFAP to label astrocytes and Congo red as a marker of fibrillar deposits. Representative images showed a correlation of GFAP-positive cells with Congo red–positive aggregates in the cortex of WT-GFP, Tg-GFP, Tg-A4, and Tg-C6T (Fig. 4*C*). Compared with age-matched WT-GFP vehicle group, the area of reactive astrocytes was greatly elevated in Tg-GFP vehicle mice (Fig. 4*D*; ^###^*p* < 0.001). In the treated groups, both C6T and A4 significantly reduced the area of reactive astrocytes, compared with littermate GFP transgenic vehicle group (Fig. 4*D*; ∗∗∗*p* < 0.001).

### C6T but not A4 reversed dendrite spine loss and synaptic aggregates

**Figure 5:**
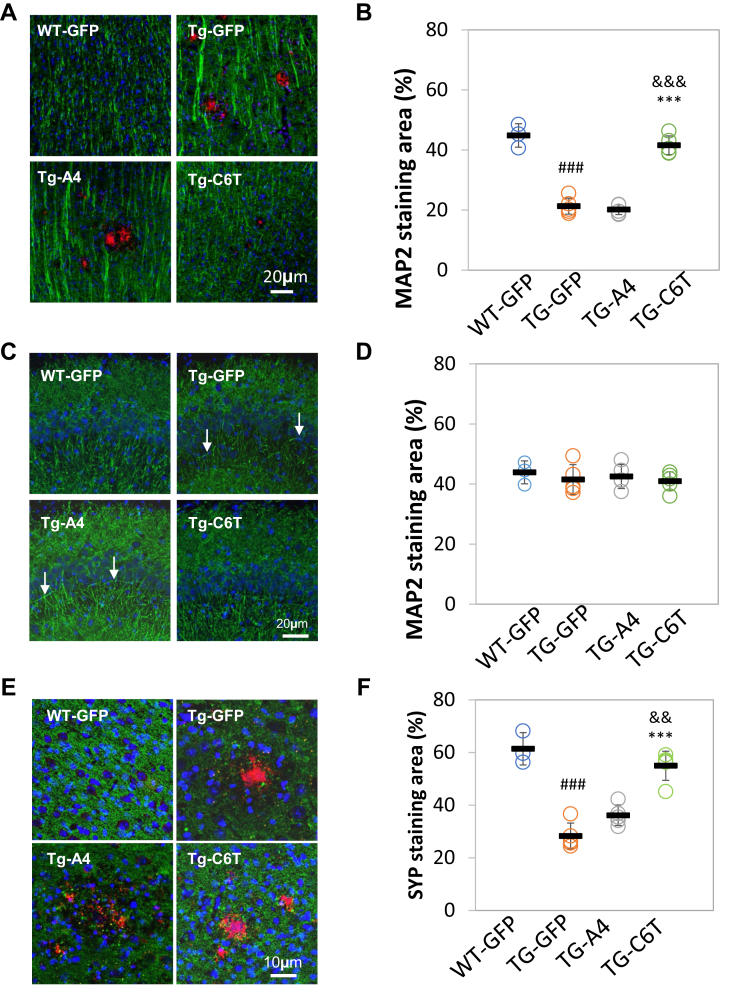
**Changes in dendritic spines and synapses in APP/PS1 mouse brain tissue.***A*, neuronal dendrites were immunostained using an antibody against MAP2 (*green*) in the cortex. *B*, the dendritic area of MAP2-positive staining was expressed as a percentage of total captured cortex regions. *C*, neuronal dendrites were visualized using an antibody against MAP2 (*green*) in CA3 region of the hippocampus. Aberrant dendritic arrangements are indicated (*arrow*). *D*, the dendritic area of MAP2-positive staining was expressed as a percentage of total captured hippocampal regions. *E*, the synapses were labeled with a specific antibody against synaptophysin (SYP, *green*). *F*, the area of SYP-positive staining was expressed as a percentage of total captured regions. In the images, Aβ deposits were labeled by antibody 6E10 (*red*). Counterstaining by 4',6-diamidino-2-phenylindole (*blue*). WT-GFP (n = 3), Tg-GFP (n = 5), Tg-A4 (n = 5), and Tg-C6T (n = 5). Statistical analysis was valued as ^###^*p* < 0.001 to age-matched WT-GFP mice, ∗∗∗*p* < 0.001 to littermate Tg-GFP vehicle group, as well as ^&&^*p* < 0.01 and ^&&&^*p* < 0.001 to littermate Tg-A4 group. MAP2, microtubule-associated protein 2.

The number of the dendrites of pyramidal neurons decreases in APP-based transgenic mouse models of AD (29, 31, 32). Dendritic density and organization were imaged using MAP-2 staining (Fig. 5*A*). Compared with WT mice, the transgenic GFP-treated mice had higher staining of MAP2 and a decreased number of MAP2-positive dendrites and spines (Fig. 5*B*; ^###^*p* < 0.001), as reported previously (22). Treatment with A4 did not rescue dendritic organization and had decreased MAP2 staining similar to the area of MAP2-positive staining in littermate Tg-GFP vehicle mice (Fig. 5*B*; *p* > 0.05). However, treatment with C6T restored the dendritic appearance and organization to levels similar to those observed in the vehicle WT mice (Fig. 5*A*). The area of MAP2-immunoreactive dendrites was also significantly elevated compared with the transgenic vehicle mice (Fig. 5*B*; ∗∗∗*p* < 0.001) and with the mice treated with A4 (Fig. 5*B*; ^&&&^*p* < 0.001).

In the CA3 region of the hippocampus, there was also considerable disorganization of the dendrites in transgenic vehicle group as well as the A4-treated mice (Fig. 5*C*; *arrowhead*). However, in the C6T-treated group, the well-organized structure of dendrites was similar to age-matched WT-GFP mice (Fig. 5*C*). Quantitatively, MAP2 staining did not show a significant loss of MAP2 expression in the hippocampus among the groups (Fig. 5*D*; *p* > 0.05). Synaptic changes in the treated mice were also characterized using synaptophysin (SYP, a presynaptic marker) and 6E10 labeling Aβ plagues. Strong staining of neurite aggregates close to 6E10-positive plaques was observed in the cortex of transgenic GFP control mice (Fig. 5*E*). There was a significant decrease in the area of SYP staining in transgenic vehicle mice, compared with WT mice (Fig. 5*F*; ^###^*p* < 0.001). Treatment with A4 did not significantly change the area of SYP-positive aggregates close to Aβ plaques when compared with the TG-GFP mice (Fig. 5, *E* and *F*; *p* > 0.05). However, treatment with C6T significantly decreased SYP aggregates (strong staining) around Aβ plaques to levels observed in the WT mice (Fig. 5*E*) and rescued SYP staining compared with levels observed in the transgenic vehicle-treated mice (Fig. 5*F*; ∗∗∗*p* < 0.001) and with the mice treated with A4 (Fig. 5*F*; ^&&^*p* < 0.01).

### C6T but not A4 greatly improves neurogenesis

**Figure 6:**
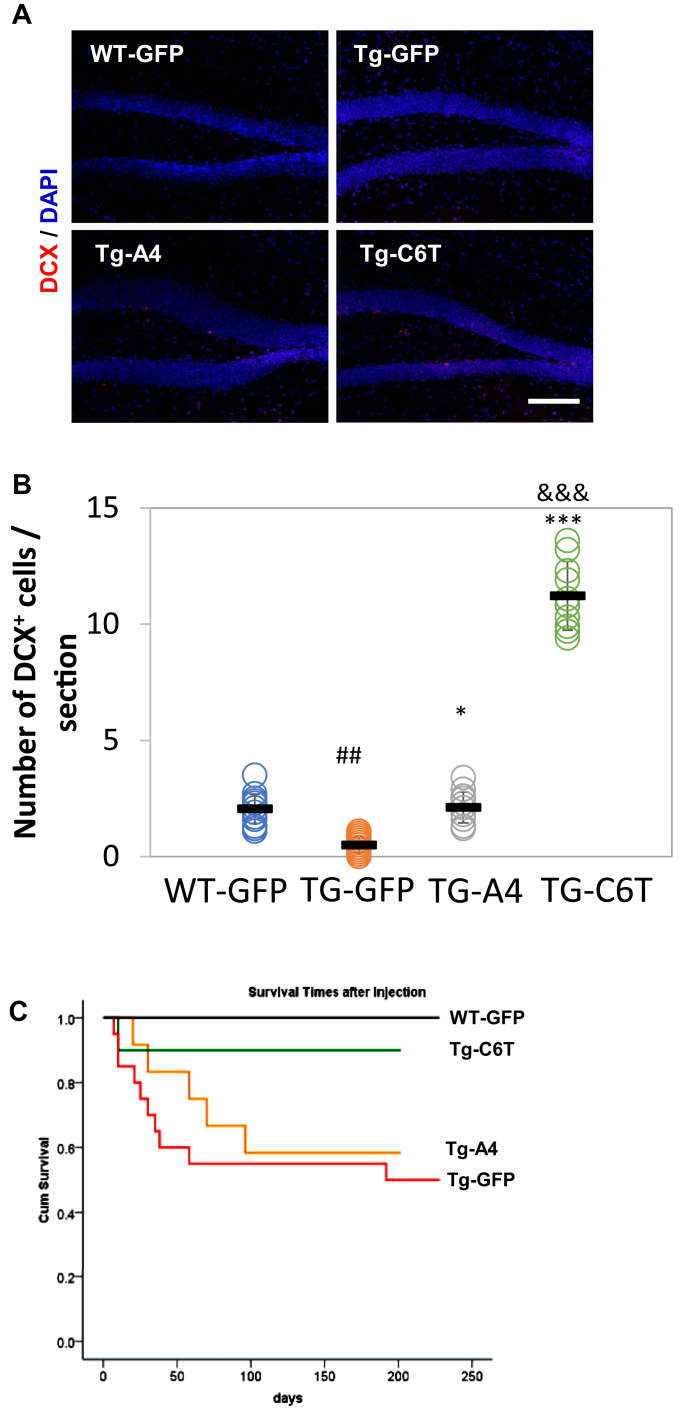
**Changes in hippocampal neurogenesis and survival rate.***A*, the newborn immature neurons in the subgranular zone of hippocampus were visualized with an antibody against DCX (*red*). Cell nuclei were counterstained by 4',6-diamidino-2-phenylindole (*blue*). *B*, the number of DCX-positive cells was counted and averaged per sections. ^##^*p* < 0.01 to age-matched WT-GFP mice, ∗*p* < 0.05 and ∗∗∗*p* < 0.001 to littermate Tg-GFP vehicle group, and ^&&&^*p* < 0.001 to Tg-A4 mice. *C*, the survival rate of the APP/PS1 mice receiving rAAV-GFP, rAAV-C6T, and rAAV-A4 with time, compared with that of age-matched WT-GFP mice as controls. At age of 2 months (the time point of viral injection), the numbers of mice injected were as follows: WT-GFP (n = 15), Tg-GFP (n = 20), Tg-A4 (n = 12), and Tg-C6T (n = 10). Seven months later, the number of mice that survived were 15, 12, 7, and 9, respectively. DCX, doublecortin.

To determine if the decrease in oligomeric Aβ levels affected the rate of neurogenesis, we tracked neurogenesis using doublecortin (DCX), a classical marker for immature neurons (33, 34). Positive staining was observed in the subgranular zone of the dentate gyrus of the hippocampus (Fig. 6*A*). We found a significant decrease in the number of DCX-positive cells of the transgenic vehicle-treated mice when compared with similarly treated WT-GFP mice (Fig. 6*B*; ^##^*p* < 0.01). With the treatment of rAAV-C6T, the number of DCX-positive cells was increased in the subgranular zone to levels higher than even the WT mice (Fig. 6*B*; ∗∗∗*p* < 0.001). Treatment with rAAV-A4 also increased the number of immature neurons compared with littermate Tg vehicle mice (Fig. 6*B*; ∗*p* < 0.05), but the levels were significantly lower than the mice receiving rAAV-C6T (Fig. 6*B*; ^&&&^*p* < 0.001).

### C6T but not A4 rescues survival in APP/PS1 mice

All mice were purchased at an age of 1 month and housed following standard Institutional Animal Care and Use Committee (Arizona State University, Tempe, AZ) protocols. WT mice receiving rAAV-GFP administration as a vehicle-negative group survived to 9 months of age with no loss of mice. APP/PS1 mice have a high mortality rate, around 40% by the age of 12 months (35). Similar to these reports, the APP/PS1 mice injected with rAAV-GFP at age of 2 months as a vehicle control group had a similar mortality rate after 7 months, with only 12 mice surviving (Fig. 6*C*). The rAAV-A4–treated mice delayed mortality but did not change the survival rate at 9 months of age. Quite impressively, the mice treated with rAAV-C6T had survival levels similar to WT with only one mouse dying at the very beginning of treatment (Fig. 6*C*).

## Discussion

We previously isolated two different scFvs, A4 and C6T, that selectively bind two different conformational variants of oligomeric Aβ (Table 1) (17, 18, 21). Here, we used these two scFvs to immunostain human postmortem AD brain tissue, where the A4 scFv-stained oligomeric Aβ variants primarily localized around the dense core of amyloid plaques. However, in an APP/PS1 AD mouse model, there was only slight staining of A4-recognized oligomeric Aβ in the diffuse plaques and similarly only slight staining of cells expressing mutant APP transgenes. Since A4 binds an Aβ oligomeric species estimated to be around the size of dodecamers, these results suggest that this population of oligomeric Aβ may be involved in Aβ oligomer-seeded fibril formation, consistent with previous reports indicating the ability of soluble high–molecular-weight Aβ species to seed Aβ plaque deposition (36, 37). In contrast, the C6T scFv, which selectively binds a small soluble toxic AD brain-derived oligomeric Aβ variant (18, 20), stained around plaques in postmortem human AD brain tissue and APP/PS1 mice. Significantly, we also observed intraneuronal staining of C6T-recognized oligomeric Aβ in brain sections of both AD samples and APP transgenic mice, as well as in the cultured cells expressing APPswe (Fig. S1). Intraneuronal oligomeric Aβ deposits were previously identified (38) and postulated to be one of the earliest events in AD pathogenesis (39, 40). Since tissue fixation for immunostaining may alter protein conformations, conclusions made from these observations must be made with caution.

Since the A4 and C6T scFvs bind different oligomeric Aβ variants that are present in different regions of human AD brain and since both scFvs were previously shown to reduce toxicity of Aβ aggregates in *in vitro* studies (17, 18, 21), here we studied the differential therapeutic effects of selectively targeting each oligomeric Aβ species *in vivo* using a mouse model of AD. Since the A4 scFv binds an oligomeric Aβ species that can be generated *in vitro* and is located extracellularly (Fig. 1) and C6T binds an oligomeric Aβ species that is generated *in vivo* and is located intracellularly (Fig. 1*C*), we can study the therapeutic benefit of targeting intracellular or extracellular toxic Aβ species. We added the ApoB gene sequence coding for the low-density lipoprotein receptor to the C-terminal region of the A4 and C6T scFvs to facilitate transport of the construct across the BBB *in vivo* (23, 41, 42). The scFv constructs were expressed in hepatic cells in an APP/PS1 AD mouse model by viral infection using a rAAV as a vector as described previously (22). The tagged scFvs are expressed by the infected hepatic cells, secreted into the blood, and then crossed the BBB into the brain. Both C6T and A4 were efficiently expressed as measured by ELISA (Fig. S2), and high levels of scFvs were detected in brain tissue indicating successful transport across the BBB essentially similar to what was reported previously (Fig. S3) (22).

While both A4 and C6T scFvs were present in the mouse brain tissue, the therapeutic benefit of the two different treatments is quite strikingly different. Loss of dendritic spine density and organization, including loss of synaptic buttons, is a common pathological feature of APP/PS1 mice (29, 31, 32). Treatment with C6T restored MAP2-labeled dendrite spine density to WT levels, whereas treatment with A4 did not alter spine density compared with vehicle-treated mice (Fig. 5, *A* and *B*). Treatment with C6T also restored synaptic density and organization including clustering of SYP-labeled synaptic buttons again to WT levels, whereas treatment with A4 did not significantly improve synaptic density or organization compared with the vehicle-treated mice (Fig. 5, *E* and *F*). Treatment with C6T also elevated neurogenesis in the hippocampus of APP/PS1 mice compared with the treatment with A4 (Fig. 6, *A* and *B*). Most dramatically, the high mortality rate of the APP/PS1 mice was rescued in the C6T-treated mice again restoring WT levels, whereas mortality was slightly delayed but essentially unchanged from the control group in the A4-treated mice (Fig. 6*C*).

Inflammation in the brain correlates strongly with neurodegeneration in AD (43) where microgliosis and astrogliosis are common features associated with Aβ deposition (44, 45). While treatment with both C6T and A4 reduced astrogliosis (Fig. 4), treatment with C6T reduced microglial activation significantly better than treatment with A4, again restoring inflammation to levels similar to that in the WT group (Fig. 4*B*). These results indicate that selectively targeting C6T-recognized oligomeric Aβ provides substantially greater therapeutic benefit toward mortality, neuronal integrity, and reducing inflammation compared with targeting A4-recognized oligomeric Aβ. However, when we analyze how the scFvs influence amyloid plaque levels, the results are quite different. Amyloid plaque levels as determined by 6E10 staining (Fig. 3, *A*–*C*) and Congo red staining (Fig. 3, *D*–*F*) showed significant reductions in the A4-treated mice but only modest reduction in the C6T-treated mice. Treatment with A4 lowered both C6T- and A4-recognized oligomeric Aβ levels in the cortex and hippocampus, whereas treatment with C6T lowered C6T-recognized Aβ variant levels to essentially WT levels but did not alter A4-recognized Aβ variant levels. The reduction in C6T levels may also be a result of virally produced C6T sterically blocking binding of the C6T scFv in the immunostains. Since treatment with C6T did not reduce plaque levels as effectively as treatment with A4, the results suggest a correlation of certain oligomeric Aβ variants (such as those recognized by A4) with the formation of Aβ plaques (36, 37, 46), whereas the smaller intracellularly brain-derived C6T-recognized oligomeric Aβ species play a more important role in synaptic toxicity consistent with previous reports (47, 48).

These results provide more evidence for the differential effects that various oligomeric Aβ species can have on neuronal health and function. Extracellularly generated oligomeric Aβ species such as those recognized by the A4 scFv seem to play an important role in seeding fibril formation and inducing astrogliosis as previously noted (46), whereas intracellularly generated oligomeric Aβ species such as those recognized by the C6T scFv are highly toxic to synapses, as previously suggested for low–molecular-weight Aβ oligomers (47, 48). There are multiple different mechanisms by which C6T may decrease levels of oligomeric Aβ and provide a therapeutic benefit. The antigen–antibody complex could be degraded by proteolysis or cleared by activated microglia in the brains, the complex may sterically interfere blocking potential toxic interactions of oligomeric Aβ, and C6T may redirect the Aβ aggregation pathway or some combination of these effects. The specific therapeutic mechanism of the C6T scFv will be the subject of further studies.

The recent promising preliminary results from a clinical trial using Aducanumab (Biogen Idec), a monoclonal antibody targeting conformation-specific Aβ aggregates, provide some promise for the strategy of targeting distinct Aβ species. A number of different Aβ oligomeric species have been identified in the recent years (49, 50), and the precise identification of the most toxic Aβ oligomer structures is still lacking (51, 52, 53, 54). The results presented here suggest that targeting specific conformational Aβ species can have profoundly different results, and that targeting intracellularly generated Aβ oligomeric species may be a very promising therapeutic strategy for treating AD.

## Experimental procedures

### Selection of C6T and A4 scFvs

We previously isolated the A4 scFv against synthetically generated oligomeric Aβ and the C6T scFv against AD brain-derived oligomeric Aβ. The A4 scFv binds an oligomeric Aβ variant with an average particle height of ∼3.6 nm and a diameter ∼10 nm (17, 21), consistent with the size of a dodecamer (13, 19), whereas C6T binds an oligomeric Aβ variant with an average particle height of ∼2.1 nm and diameter of ∼5 nm, consistent with the size of trimeric/tetrameric Aβ (18) (Table 1).

### Construction of rAAV vectors

As described previously (22), the complementary DNA constructs of the C6T and A4 scFv genes were amplified by PCR and then cloned into pFBAAVCAGmcsBgHpA vector (G0345; Viral Vector Core Facility). The FLAG tag was placed at the C-terminal region of the scFv for use as a marker. The vectors AAV2/8-containing plasmids pAAV-scFv were generated through triple transfection into HEK293 cells (Viral Vector Core Facility). After cellular infection, the vectors encoding either the A4 or C6T scFv along with C-terminal ApoB tag were verified to bind the respective conformations of Aβ aggregates. A control rAAV-encoding GFP without ApoB or FLAG was prepared similarly to the scFv constructs. The release of viral particles containing the vector genomes was measured, and the titers of rAAV virions were determined by a quantitative dot-blot assay (Viral Vector Core Facility).

### Human samples

Human brain tissues were obtained from Dr Thomas Beach, the Brain and Body Donation Program at Banner/Sun Health Research Institute (http://www.brainandbodydonationprogram.org) (55, 56). The average postmortem interval was less than 3 h. The average age of the AD and ND subjects was 76.8 ± 8.8 and 85.2 ± 8.5 years old, respectively. The neuropathological characteristics are shown in Table 2. The postmortem AD and cognitively normal controls (ND) utilized were confirmed pathologically. The neocortex of superior frontal cortex was sectioned with 40 μm, and immunolabeling was described following immunofluorescence staining.

**Table 2:** The pathological profiles of human subjects from the brains with AD and controls

Case ID	Gender	Age (year)	Neuropathological diagnosis	MMSE	Postmortem interval (h)	Disease duration (year)	ApoE
97-15	M	77	AD	NA	2.33	12	3/4
00-37	M	81	AD	6	3	13	3/4
03-06	F	89	AD	6	2.33	9	3/4
03-07	M	67	AD	13	2	8	3/4
04-33	M	70	AD	6	2.33	11	3/4
01-31	M	81	ND	25	2.75		3/3
01-46	F	90	ND	27	3		3/3
03-63	F	83	ND	29	3.25		3/3
07-04	M	97	ND	NA	1.5		2/3
07-11	F	75	ND	NA	2.75		3/4

ApoE, apolipoprotein E; F, female; M, male; MMSE, Mini-Mental State Examination.

### Animals

One-month-old female APP/PS1 mice (Mutant Mouse Resource and Research Center 34832) and age-matched female WT mice were purchased from Jackson Laboratory. The transgenic mouse is generated on a genetic background C57BL/6J expressing Swedish mutation KM670/671NL of human APP (APPswe) and a presenilin 1 lacking exon 9 (PSEN1dE9) (27). After 6 to 8 weeks, the transgenic mice showed cerebral amyloidosis and amyloid-associated pathologies, including dystrophic synaptic buttons, robust gliosis, and increase in microglia number and activation (29). All protocols for animal use here were approved by the Institutional Animal Care and Use Committee. Animals were treated in accordance with good animal practice following National Institutes of Health requirements.

### Administration of rAAV-scFvs

Two-month-old APP/PS1 mice were randomly assigned into one of three groups: two test groups receiving either rAAV-C6T (n = 10) or rAAV-A4 (n = 12) or a positive vehicle control group rAAV-GFP (n = 20). The negative control received rAAV-GFP in age-matched WT (n = 15). All the mice were subjected to a single intraperitoneal injection of rAAV-scFv or rAAV-GFP at a dosage of 3.0 × 10^10^ vg/mouse essentially as described previously (22).

### Tissue harvest

Seven months after the injection, surviving mice were euthanized with isoflurane and perfused with 0.1 M phosphate buffer including 10 U of heparin (AK3004; Akron Biotech). Survival numbers for each group were as follows: Tg-C6T (n = 9), Tg-A4 (n = 7), Tg-GFP mice (n = 12), and WT (n = 15). The mouse brain tissues were quickly harvested. The right hemispheres were stored at −80 °C for biochemistry, and the left hemispheres were fixed in 4% (w/v) paraformaldehyde for histology.

### ELISA

Samples were homogenized using homogenization buffer, which contains 1% Nonidet P-40 (Calbiochem) and protease and phosphatase inhibitor cocktails (Roche). The homogenate was centrifuged at 14,000 rpm for 20 min. The protein concentration of supernatants was measured with a Pierce bicinchoninic acid protein assay kit (Thermo Scientific) as total protein values. The sandwich ELISA was performed essentially as described previously (25, 26). Briefly, the captured scFv was immobilized to the wells of a high-binding 96-well ELISA plate (Costar), and any unbound sites were blocked with 2% milk in PBS. Homogenized samples were added to the wells for 2 h of specific binding. After washing, homogenized samples were incubated for 2 h at 37 °C followed by 200 ng/ml of a 40 mM carboxyl-biotinylated detection phage. Any bound biotinylated phage was identified using an avidin–horseradish peroxidase antibody. Following addition of the SuperSignal ELISA Femto Maximum Sensitivity Substrate (Thermo Scientific), signal intensities were quantified using the Wallac Victor^2^ microplate reader. After each incubation step, the wells were washed 3 to 4 times with 0.1% PBS-Tween20 to reduce nonspecific binding.

### Chromogenic 3,3′-diaminobenzidine immunostaining

The brain sections were prepared as described previously (22, 55). In brief, the brain tissues were sagittally sectioned 30 μm thick with a Cryostat (CM3000; Leica). Immunostaining was performed as described previously (22, 34, 57). Briefly, after blocking nonspecific protein binding, the sections were then incubated with a monoclonal antibody 6E10 (catalog no.: SIG-39320; 1:2000 dilution; Covance) and monoclonal antibody against GFAP (catalog no.: SMI-22R; 1:5000 dilution; Covance). Then sections were incubated with biotinylated secondary antibody of horse antimouse IgG. Following washing, the Vectastain kit (Vector Laboratories) was applied. Samples were visualized using 3,3′-diaminobenzidine as a substrate (Vector Laboratories). The sections were processed deleting primary antibody as negative controls and counterstained with hematoxylin (Sigma–Aldrich).

### Congo red staining

As described previously (22), the fibrillar deposits were visualized with histological staining of Congo red (C6T277; Sigma–Aldrich). Prior to incubation, the solution was filtered. After the incubation of Congo red for 20 min, the sections were counterstained with hematoxylin to show cell nuclei (Sigma–Aldrich).

### Immunofluorescent staining

Immunofluorescent staining of oligomeric Aβ by C6T and A4 was performed as follows. In brief, the human or mouse brain sections were incubated with purified scFv antibody A4 and C6T (1 μg/ml) at 4 °C overnight. Then, a rabbit anti-cMyc antibody (C3956; 1:1000 dilution; Sigma–Aldrich) was used to detect the cMyc tag on the C6T and A4 scFvs. Images were double stained with antibody 6E10 to label Aβ (SIG-39320; 1:2000 dilution; Covance) or an antibody against MAP2 as a neuronal marker (MMS-485P; 1:500 dilution; Covance). Sudan black was used for quenching nonspecific fluorescence of intracellular lipofuscin. A control using secondary antibody against Myc tag without the corresponding primary scFv was also performed (Fig. 1). Microglial cells were visualized with rabbit anti-ionized calcium-binding adaptor molecule 1 (Iba1) antibody (catalog no.: 019-19741; 1:500 dilution; Wako). The synapses were immunostained using a rabbit antibody against SYP (Santa Cruz; 1:100 dilution), and newborn neurons in the hippocampus were labeled with rabbit anti-DCX (Ab18723; 1:500 dilution; Abcam). Fluorescent-conjugated secondary antibodies were used to visualize the target structures. The endogenous lipofuscin was blocked with 0.3% Sudan black, and cell nuclei were labeled with 4',6-diamidino-2-phenylindole (Electron Microscopy Sciences).

### Quantification of immunostaining structures

The quantification of immunostaining structures was performed as described previously (22). A microscope BX51T-PHD-J11 (Olympus) was utilized to capture images. The Image J analysis software (National Institutes of Health) was employed to analyze the structures of interest. A semiautomated red–green–blue color threshold was set to determine the optimal threshold settings. We subtracted the background counterstain with a deconvolution method to determine the specific mean immunostaining. An average value of immunostaining areas was expressed as a percentage of total area in the cortex or the hippocampus. An average value was determined for each group, and data were expressed as mean ± SD.

### Statistical analysis

Statistical analyses were performed as described previously (22). We used SPSS 13.0 software (SPSS, Inc). In order to determine the significant intergroup difference, two-way repeated measures of ANOVA and Student–Newman–Keuls *q* test or Dunnet *t* test were applied. A statistical significance was considered as the value of *p* < 0.05.

## Data availability

Data are available upon request. Please contact Dr Michael Sierks (sierks@asu.edu), Arizona State University.

## Conflict of interest

M. Sierks is a cofounder of Studio Biotherapeutics.
