# Supplementary material for: A conformation-specific antibody against oligomeric β-amyloid restores neuronal integrity in a mouse model of Alzheimer's disease
Source: J Biol Chem. 2021 Jan 9;296:100241. doi: 10.1074/jbc.RA120.015327 (PMC7948963; doi:10.1074/jbc.RA120.015327)
Supplement: Supplemental Figures [file mmc1.pdf]

Results

Intracellular localization of C6T-recognized oligomeric variants

To further confirm that C6T-recognized oligomeric Aβ variants were intracellularly located in human and APP/PS1 mouse brain tissue (**Fig. 1, 2**), we cultured HEK293 cells overexpressing human Aβ and double immunostained with scFvs and Aβ specific antibody 6E10. Generation of A4- or C6T-recognized Aβ species were not observed in HEK293 cells without transfection of mutated human APP (**Fig. S1A, B**). A4-recognized Aβ variants were not observed in HEK293 cells overexpressing APPswe (**Fig. S1A**), however, oligomeric Aβ variants recognized by C6T were observed in the cytoplasm of the HEK293 overexpressing mutated human APP (**Fig. S1B**). The C6T scFv co-stained with Aβ peptide labeled by 6E10 confirmed the presence of oligomeric Aβ. The intracellular localization of C6T recognized Aβ variants is consistent with our observations of human AD and APP/PS1 mouse brain tissue.

Viral infection exclusion of brains receiving rAAV-scFv

To verify that viral infection and subsequent scFv expression was occurring in hepatic cells and not brain cells, we analyzed liver and brain tissue samples essentially as described previously (1). While rAAV capsid AAV2 was visualized in all liver tissues of rAAV2/8 vector groups following intraperitoneal (IP) delivery for 9 months (**Fig. S2A)**, no AAV2 staining was observed in brains of the same mice receiving rAAV (**Fig. S2B)**. Next, we observed GFP expression directly under fluorescence confocal microscopy in the liver sections of the mice receiving rAAV-GFP, rAAV-A4 and rAAV-C6T. A strong fluorescent expression of GFP was seen in the hepatic cells receiving rAAV-GFP but not in the results obtained with AAV capsid staining, no GFP expression was observed in brain samples of mice infected with rAAV-GFP, rAAV-A4 or rAAV-C6T (**Fig. S2D)**. These results indicate that active rAAVs expression was taking place in liver cells but not in brain cells as reported earlier (1).

ScFv expression in liver and brain tissue after infection with rAAV

Next, we verified that the scFvs were expressed by infected liver cells and transported through into the brain by the ApoB tag. The liver tissue sections were immunostained with an antibody against FLAG, a marker for the A4 and C6T scFvs. As expected, FLAG was visualized in the hepatic cells of the APP/PS1 mice administered rAAV-A4 and rAAV-C6T mice but not in that of WT and Tg mice receiving rAAV-GFP (**Fig. S3A**). Similarly, the immunostaining of brain sections showed a strong labeling of FLAG in the brains of APP/PS1 mice receiving rAAV-A4 and rAAV-C6T but negative staining in the WT and APP/PS1 mice receiving rAAV-GFP (**Fig. S3B)**, indicating that the ApoB tag on the scFvs could facilitate the transport across the BBB. Levels of scFvs in the respective brain tissues were determined by ELISA. A commercial protein containing multiple tag markers including FLAG was utilized as a standard. Brain levels of the tag FLAG were measured and normalized to pg/mg of the brain tissues. ELISA results showed high expression of scFv levels in the cortex tissue of the mice transfected with rAAV-A4 and rAAV-C6T but not in the WT-GFP and Tg-GFP mice (**Fig. S3C**). These results indicate successful expression of scFv by infected liver cells and transport across the BBB into the brain, essentially as reported earlier(1) .

Both scFv-A4 and C6T Administration did not change APP Metabolism in APP/PS1 Mice

To exclude the possible effects of scFv administration on APP processing as we previously observed with this mouse line when administering an antibody designed to modify APP processing (1), APP cleavage fragments were analyzed by Western blot (Fig. S4). There were not any obvious differences in expression levels of the APP cleavage enzyme BACE1 between WT and transgenic mice with or without scFv application. The cleavage fragment by BACE1, human soluble β APP Swedish mutation (sAPPβ-sw) was expressed in human APP transgenic mice but not WT. The levels of sAPPβ-sw, however, were not significant different in the transgenic mice with or without scFv application (Fig. S4A, B). To further confirm the APP amyloidogenic processing, the cleavage of APP C-terminal fragment was immunoblotted. We did not find any significant expression changes of APP as well as α, β fragments of C-terminals (CTFα, and CTFβ) among Tg-GFP, Tg-A4 and Tg-C6T, but no visible expression of APP fragments in WT-GFP (Fig. S4C, D).

Materials and Methods

Cell culture and immunofluorescence

HEK293 cells stably transfected with the Swedish mutation (K595N/M596L) of human amyloid precursor protein (APPswe) (gift from Dr. Hailan Yao from Roskamp Institute, Sarasota, FL) were maintained in Dulbecco's modified Eagle's medium with 10% fetal bovine serum (FBS) supplemented with G418 sulfate 0.6mg/ml (Geneticin, Catalog:10131035, Gibco, Thermo Fisher Scientific). Untransfected HEK293 cells were cultured in 10% FBS without G418, as controls. The cells were seeded on a glass chamber with 0.5% FBS for 24 h and then fixed with 4% paraformaldehyde for 10 min. The nonspecific staining was blocked with 10% goat serum for 30 min. The cells were incubated with purified A4 or C6T scFv. Then antibodies against c-Myc tagged scFv (Sigma, 1:1000) and Aβ1-17 (6E10, Covance, 1:1000) were applied. Total Aβ and the C6T- and A4-recognized oligomeric variants of Aβ were visualized with fluorescent-labeled secondary antibodies (1:1000, Invitrogen, Thermo Fisher Scientific).

Immunofluorescent staining

As described previously(1), sections of liver and brain tissue samples were incubated with antibodies against adeno-associated virus capsid VP3 (AAV2, Novus Biologicals, Centennial, CO) and against FLAG (Sigma-Aldrich, St. Louis, MO). Sudan black was applied to block the non-specific fluorescence of the endogenous lipofuscin. Cell nuclei were labeled with DAPI. The localization of GFP was directly observed and the images were captured by the confocal Leica TCS SP5 (Biodesign Institute, Arizona State University, AZ).

ScFv levels in brain tissue

The levels of both A4 and C6T scFvs were measured as described previously (1). In brief, the fresh cortical tissues were harvested and was homogenized in homogenization buffer. A designed fusion protein, Multiple Tag, containing the tag-FLAG sequence DYKDDDDK (Catalog: M0101, GenScript, Piscataway, NJ), with a molecular weight of ~40 kDa on SDS-PAGE was used as a standard for calculating scFv concentration. The molecular weight of the tagged A4 and C6T scFvs were estimated as 30 KDa. A monoclonal antibody against tag FLAG (Sigma-Aldrich St. Louis, MO) was applied. The protein assay was performed with ELISA method. The readout values of scFv proteins were normalized to pg/mg of brain tissues.

Immunoblot Analysis

The immunoblot analysis was performed as described previously (1). In brief, the mouse cortex tissue was homogenized in PBS buffer (Sigma-Aldrich, St. Louis, MO), supplemented with 1% Nonidet P-40 (Calbiochem, Billerica, MA), and protease and phosphatase inhibitor cocktails (Roche, Pleasanton, CA). The homogenate was centrifuged at 14,000 rpm for 20 min. The protein concentration was measured with a BCA protein assay kit (Pierce Biotechnology, Rockford, IL). The supernatants (50 μg) mixed with 2× Laemmli sample buffer (Bio-Rad, Hercules, CA) with 0.001% bromphenol blue were heated at 75°C for 10 min and loaded to 8% SDS-PAGE. The separated proteins were electrotransferred to nitrocellulose membranes (Millipore, Bedford, MA). The membranes were blocked with 5% dry milk and were incubated with primary antibodies overnight: rabbit anti-N-terminal BACE1 (B0681, clone: 46-62, Sigma-Aldrich), mouse anti-human soluble β APP (Swedish mutation) (sAPPβ, catalog: 10321, Clone: A61, IBL-America). C-terminal fragment of APP were detected using rabbit polyclonal antibody (catalog: 171610, clone: 751–770, Sigma Aldrich). The membrane was then incubated with the secondary antibodies, goat anti-mouse or rabbit IgG conjugated with horseradish peroxidase (Santa-Cruz Biotechnology, Santa Cruz, CA) and visualized by an enhanced DAB system (Sigma-Aldrich) following the manufacturers' instructions. Semi-quantification analysis was performed using a Versadoc XL imaging apparatus (Bio-Rad). β actin (catalog: A1978; clone AC-15, Sigma-Aldrich) levels were used as loading controls.

Reference

1. He, P., Xin, W., Schulz, P., and Sierks, M. R. (2019) Bispecific Antibody Fragment Targeting APP and Inducing alpha-Site Cleavage Restores Neuronal Health in an Alzheimer's Mouse Model. *Mol Neurobiol* **56**, 7420-7432

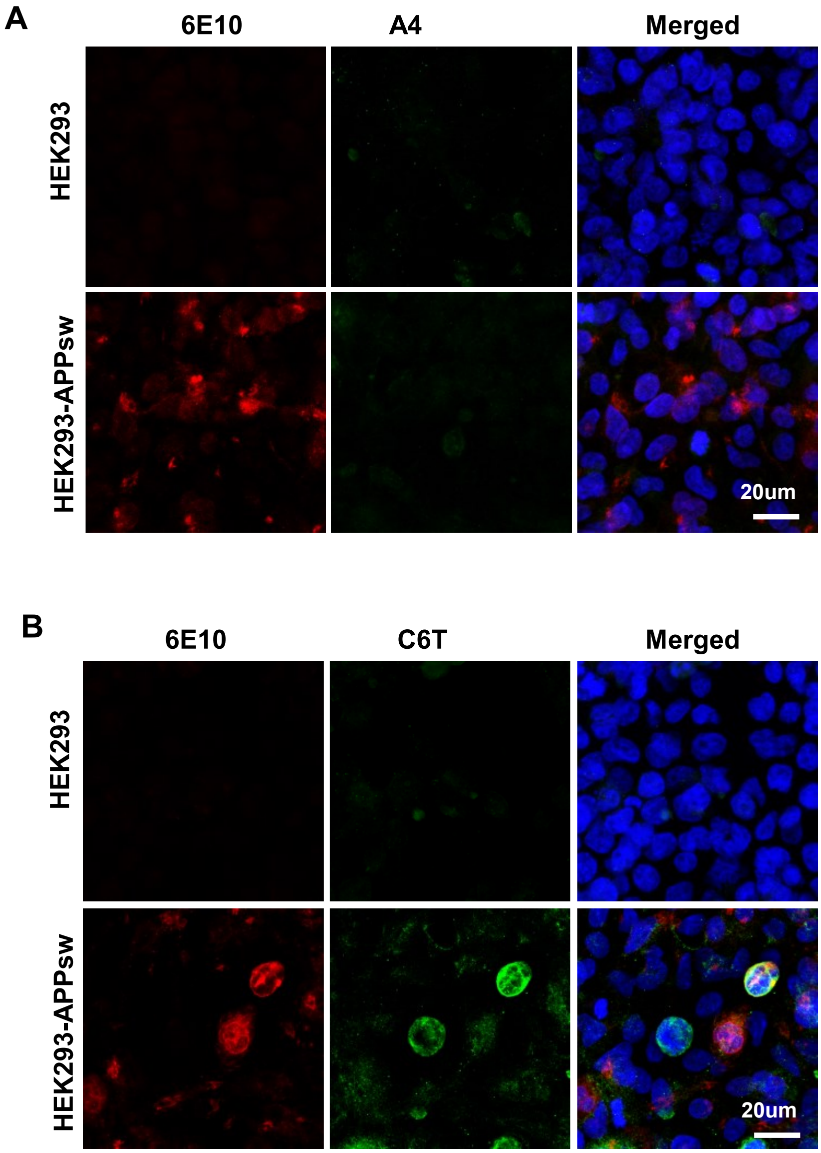

Figure S1. Cellular location of scFv-recognized oligomeric variants. HEK293 cells and HEK293 cells expressing the human APPswe mutation were incubated in DMEM with 0.5% FBS for 24 h and fixed with 4% PFA for 10 min. **A.** Oligomeric Aβ species recognized by A4 were immunolabeled with an antibody against the c-Myc tag (green) on the scFv, and by a monoclonal antibody 6E10 (red) against Aβ. **B.** Oligomeric Aβ species recognized by C6T were immunolabeled with an antibody against c-Myc (green), and Aβ by monoclonal antibody 6E10 (red). Nuclei were counter stained with DAPI (blue). Bar: 20μm.

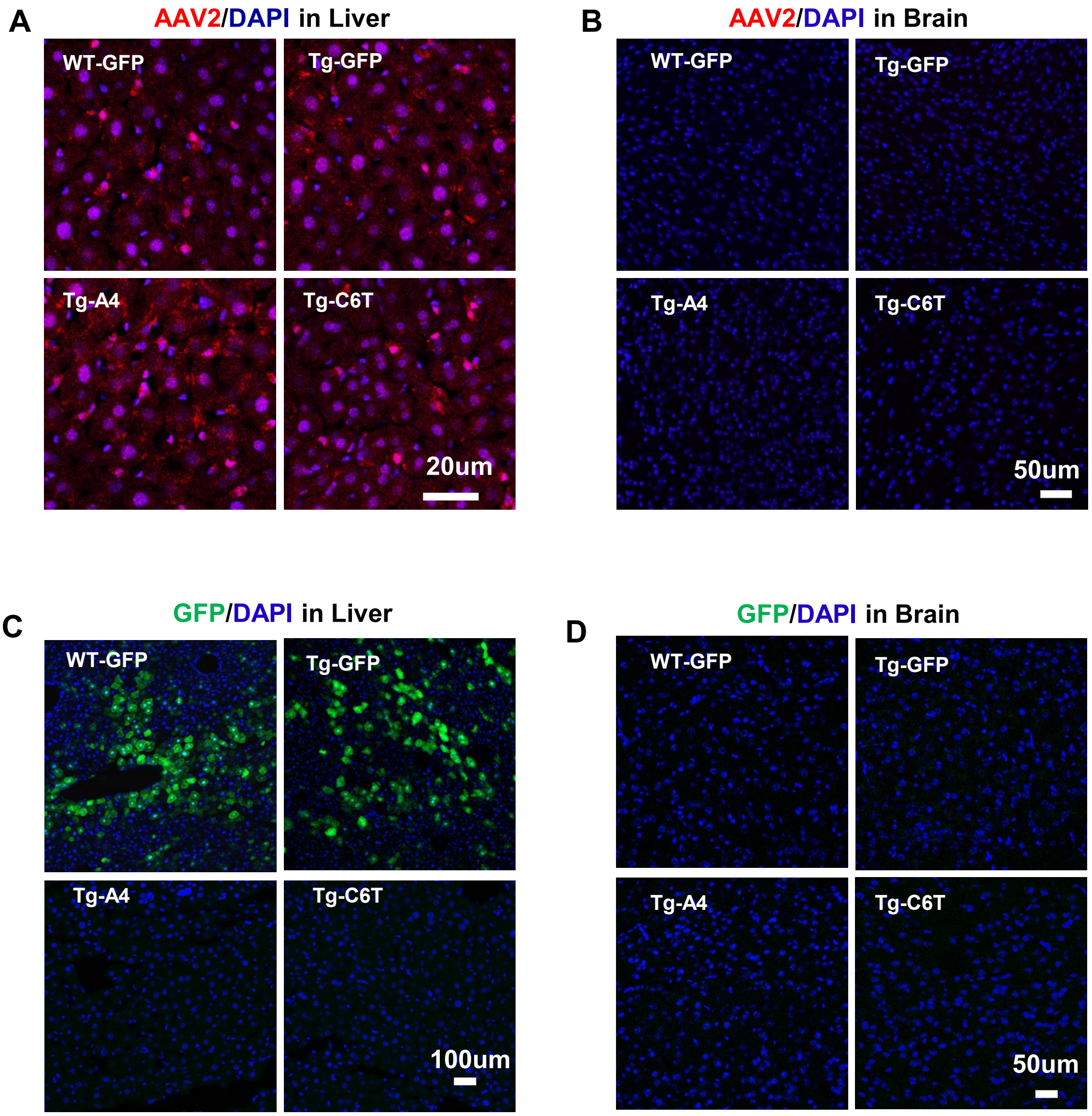

Figure S2. Viral infection in brains receiving rAAV. A. Active rAAVs were stained with an antibody against rAAV capsid AAV2 (red) in the hepatic cells of the mice administered rAAV intraperitoneally. **B.** The rAAV capsid AAV2 was immunostained (red) in brain sections of the mice receiving rAAV. **C.** The green fluorescence of GFP expression was observed in the liver tissues of mice injected with rAAV-GFP, rAAV-A4 and rAAV-C6T. **D.** No protein expression of GFP (green) was observed in the brain sections of mice injected with rAAV-GFP, rAAV-A4 and rAAV-C6T. Cell nuclei were counterstained with DAPI (blue).

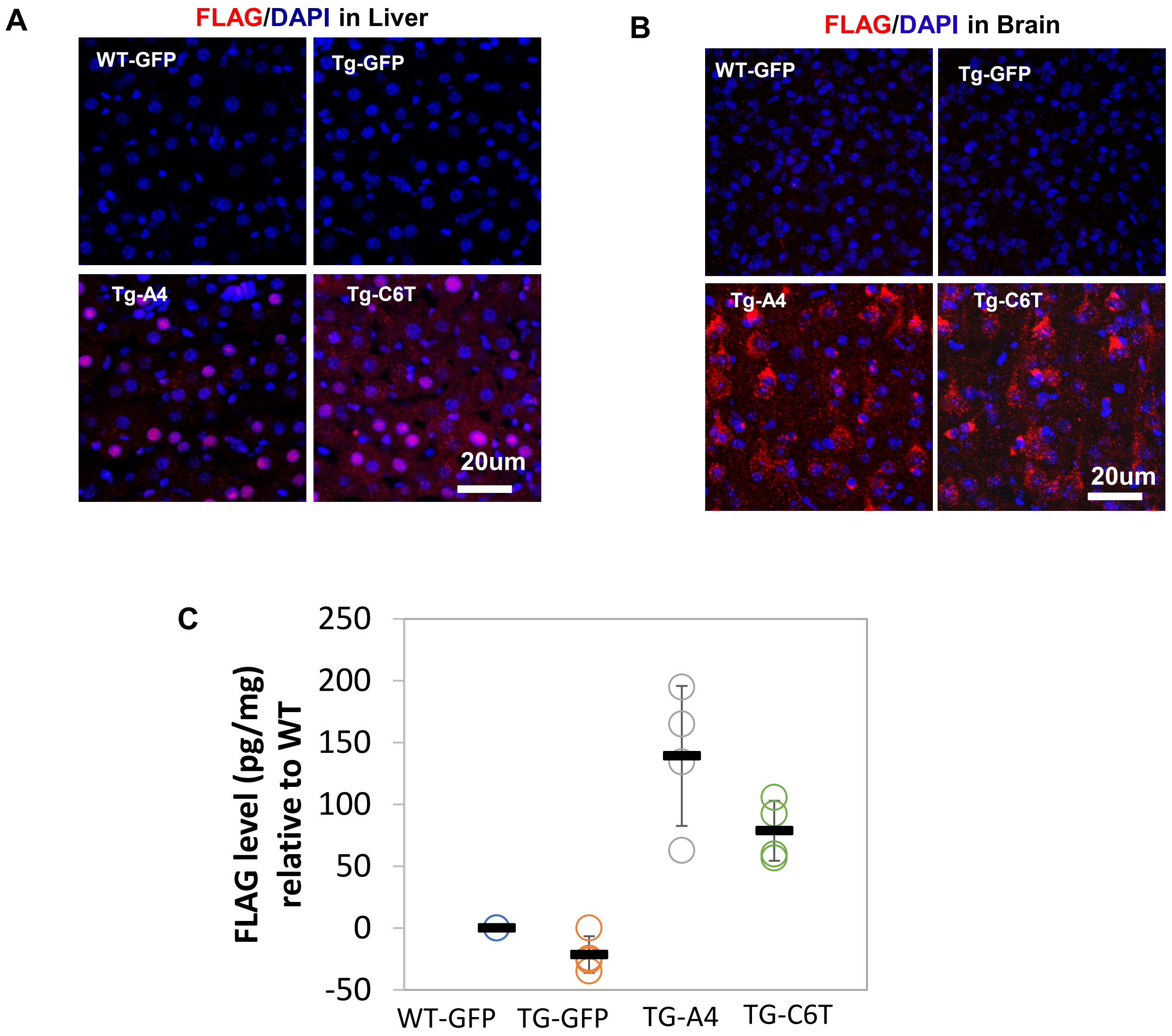

Figure S3. Delivery of scFv to brains receiving rAAV. A. FLAG immunostaining (red) in the hepatic tissues of the mice receiving rAAV-GFP, rAAV-A4 or rAAV-C6T. **B.** FLAG immunostaining (red) in the brain tissues of the mice receiving rAAV-GFP, rAAV-A4 or rAAV-C6T. **C.** ELISA measured scFv concentration by rabbit anti-tag FLAG antibody. Data were expressed as pg/mg of the brain tissue receiving the application of rAAV-GFP, rAAV-A4 or rAAV-C6T. n = 5 in each group.

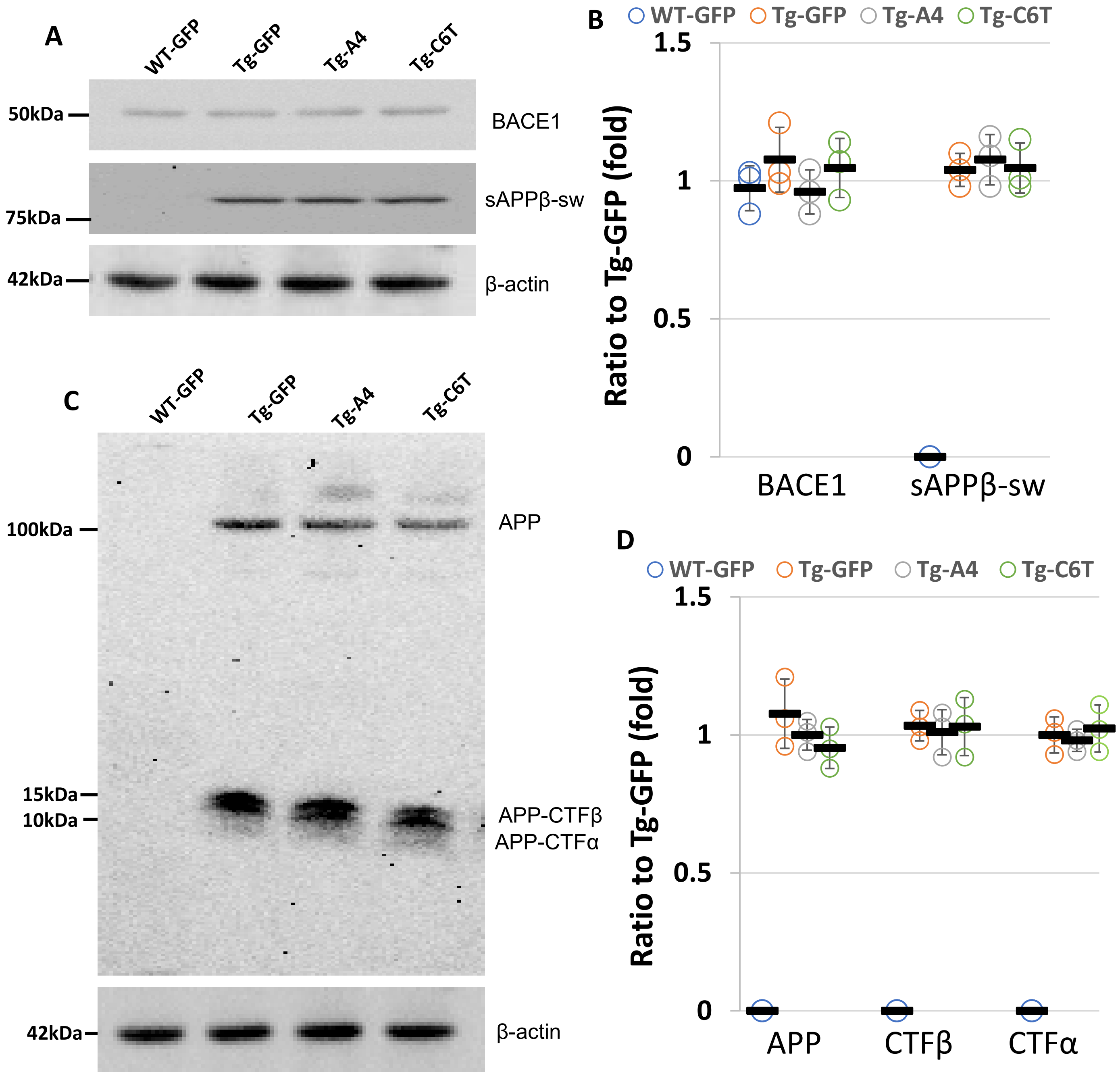

Figure S4. APP amyloidogenic proceeding in the brains of APP/PS1 mice. A. Representative expression showing the expression of BACE1 as well as sAPPβ-sw in the cortex by Western blot. B. Normalized to age-matched Tg-GFP, semi-quantification showed the total amount of BACE1 and sAPPβ-sw in the cortex among the mice administering WT-GFP, Tg-GFP, Tg-A4 and Tg-C6T. $n = 5$ each group, $p > 0.05$. C. Representative image showing the expression of human APP and its C-terminal fragments, CTFα, and CTFβ, in the cortex by Western blot. D. Normalized to age-matched Tg-GFP, semi-quantification showed the amount of APP and CTFs in the cortex among the mice administering Tg-GFP, Tg-A4 and Tg-C6T. $n = 5$ each group, $p > 0.05$.
